# Spasmolytic and Uroprotective Effects of Apigenin by Downregulation of TGF-β and iNOS Pathways and Upregulation of Antioxidant Mechanisms: In Vitro and In Silico Analysis

**DOI:** 10.3390/ph16060811

**Published:** 2023-05-30

**Authors:** Saima, Irfan Anjum, Aisha Mobashar, Shah Jahan, Saima Najm, Hiba-Allah Nafidi, Yousef A. Bin Jardan, Mohammed Bourhia

**Affiliations:** 1 Department of Pharmacology, Faculty of Pharmacy, The University of Lahore, Lahore 54000, Pakistan; 2 Department of Immunology, University of Health Sciences Lahore, Lahore 54600, Pakistan; 3 Department of Pharmacy, Lahore College of Pharmaceutical Sciences, Lahore 54000, Pakistan; 4 Department of Food Science, Faculty of Agricultural and Food Sciences, Laval University, Quebec City, QC G1V 0A6, Canada; 5 Department of Pharmaceutics, College of Pharmacy, King Saud University, Riyadh 11481, Saudi Arabia; 6 Laboratory of Chemistry and Biochemistry, Faculty of Medicine and Pharmacy, Ibn Zohr University, Laayoune 70000, Morocco

**Keywords:** apigenin, pro-inflammatory cytokine, antioxidant, interstitial cystitis, bladder relaxant

## Abstract

Apigenin is a phytochemical obtained from *Chamomilla recutita.* Its role in interstitial cystitis is not yet known. The present study is aimed at understanding the uroprotective and spasmolytic effects of apigenin in cyclophosphamide-induced interstitial cystitis. The uroprotective role of apigenin was analyzed by qRT-PCR, macroscopic analysis, Evans blue dye leakage, histological evaluation, and molecular docking. The spasmolytic response was measured by adding cumulative concentrations of apigenin to isolated bladder tissue pre-contracted with KCl (80 mM) and carbachol (10^−9^–10^−4^) on non-incubated and pre-incubated tissues with atropine, 4DAMP, methoctramine, glibenclamide, barium chloride, nifedipine, indomethacin, and propranolol. Apigenin inhibited pro-inflammatory cytokines (IL-6, TNF-α and TGF 1-β) and oxidant enzymes (iNOS) while increasing antioxidant enzymes (SOD, CAT, and GSH) in CYP-treated groups compared to the control. Apigenin restored normal tissue of the bladder by decreasing pain, edema, and hemorrhage. Molecular docking further confirmed the antioxidant and anti-inflammatory properties of apigenin. Apigenin produced relaxation against carbachol-mediated contractions, probably via blockade of M_3_ receptors, K_ATP_ channels, L-type calcium channels, and prostaglandin inhibition. While the blockade of M_2_ receptors, K_IR_ channels, and β-adrenergic receptors did not contribute to an apigenin-induced spasmolytic effect, apigenin presented as a possible spasmolytic and uroprotective agent with anti-inflammatory, antioxidant effects by attenuating TGF-β/iNOS-related tissue damage and bladder muscle overactivity. Thus, it is a potential agent likely to be used in treatment of interstitial cystitis.

## 1. Introduction

Interstitial cystitis (IC) is an inflammatory disorder often accompanied by suprapubic pain and increasing voiding rate and frequency [1]. Patients with IC typically complain of sexual and sleep disorders, emotional stress, depression, and nervousness. Interstitial cystitis is known to depreciate the value of life for millions of people. Females are more likely to develop this condition than males [2]. Various models have been developed to simulate interstitial cystitis, and cyclophosphamide (CYP)-induced IC is among the most extensively used models [3]. In the liver, acrolein, a metabolite of cyclophosphamide, is produced and damages, leading to symptoms of IC [4]. Several mediators like interlukin-6 (IL-6), interlukin-1(IL-1), tissue necrosis factor -α (TNF-α), transforming growth factor 1-β (TGF 1-β), inducible nitric oxide synthase (iNOS), and catalase (CAT) are involved in the pathophysiology of IC [5,6]. These are produced as a result of urothelial damage caused by acrolein accumulation. Mesna (2-mercaptoethanesulfonate) is co-administered with cyclophosphamide, as it has the ability to bind with acrolein. Bound acrolein is unable to concentrate in urothelium, leading to inhibition of oxidative stress, inflammation, and necrosis [7]. It is, however, observed that preventive mesna therapy leads to bladder injury, edema, hemorrhage, and inflammation. New alternative drugs are being tested to prevent CYP-induced IC [8]. Pretreatment with phytochemicals that increase antioxidant enzyme levels and decrease pro-inflammatory cytokines have shown promising response earlier in CYP-induced IC [5].

Inflammatory response in the bladder is in part due to the release of interleukins, the cytokines secreted from ulcerated bladder epithelium. The interleukins attract lymphocytes, which are responsible for the pathological aspects of disease [9]. The bladder wall permeability is altered due to disruption of the GAG (glycosaminoglycan) layer, thus increasing its permeability. The urinary solutes accumulate in the stoma, causing inflammation, infiltration of mast cells, and nociception nerve sensitization. TNF-α increases the permeability of epithelial cells. It induces the production of pro-inflammatory cytokine IL-6. The production of cytokines is also enhanced by TGF 1β. It activates macrophages, which is important in developing inflammation in later stages of disease [10,11]. IL-6 also alters detrusor muscle function, which is manifested by decreased urine-holding capacity and frequent urination. Antioxidants like GSH, CAT, and SOD defend cells from radical assault, as they maintain the redox potential of cells. Inducible NO synthase (iNOS) expression has been upregulated in patients with IC. Free radicals cause damage and inflammation by destroying cellular architecture [12]. Muscarinic receptors are abundantly found in bladder tissue. In inflammatory conditions, muscarinic responses are transformed, and detrusor hyperexcitability is reported in cystitis [13,14].

Recently, alternative medicines have been utilized for treating various serious ailments. Herbal extracts have rich antioxidant and anti-inflammatory compounds that exhibit a plethora of activities [15]. Phytochemicals have been studied for their potential role in cyclophosphamide-induced interstitial cystitis and have proven helpful, thus gaining immense attention [16].

Apigenin is a plant flavonoid extracted from *Chamomilla recutita* (L.) [17]. In several experimental settings, it has been effective in treating diabetes, inflammation, and cell apoptosis [18]. It is an antioxidant that was used in the treatment of hepatocellular carcinoma in the Wistar albino rat model [19]. Its role has also been evaluated in conditions like osteoarthritis, nephrolithiasis, urolithiasis, hypertension, Alzheimer’s disease, anxiety, and depression [20]. However, its potential in the treatment of interstitial cystitis has not yet been studied. Thus, the present study was designed to determine the spasmolytic and uroprotective roles of apigenin in cyclophosphamide-induced interstitial cystitis.

## 2. Results

### 2.1. Effect of Apigenin on the Expression of Inducible Nitric Oxide Synthase (iNOS), Glutathione Reductase (GSH), Superoxide Dismutase (SOD), and Catalase (CAT)

**Figure 1:**
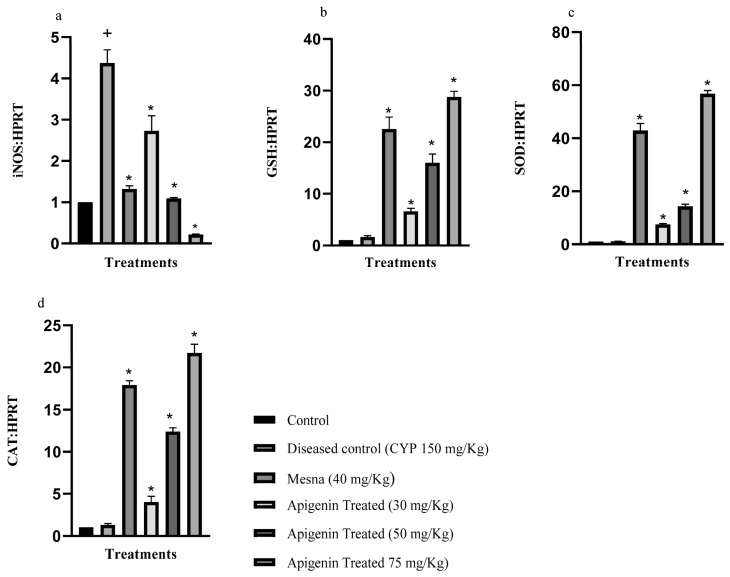
Reverse transcription polymerase chain reaction of oxidant and antioxidant enzymes. Relative gene expression of (**a**) inducible nitric oxide synthase (iNOS), (**b**) glutathione reductase (GSH), (**c**) superoxide dismutase (SOD), (**d**) catalase (CAT), mRNA as normalized to Hypoxanthine-guanine phosphoribosyltransferase (HPRT) mRNA. The values were presented as mean ± standard error of mean *n* = 3. * *p* < 0.0001 relative to the diseased control group. ^+^ *p* < 0.0001 relative to the control. One-way ANOVA followed by Tukey’s multiple comparison test was applied. SEM was represented by vertical bars.

The mRNA expression levels of iNOS, GSH, SOD, and CAT were measured. In the diseased control, oxidant enzyme iNOS levels were significantly (*p* < 0.05) greater compared to the control group. The apigenin treatment (30, 50, and 75 mg/kg) significantly (*p* < 0.05) reduced iNOS mRNA expression compared to the diseased. The mRNA expression of antioxidant enzymes GSH, CAT, and SOD was reduced in the CYP-treated group. Apigenin, in increasing dose, restored this mRNA level significantly (*p* < 0.05) compared to the diseased group. Apigenin-treated (75 mg/kg) rats showed higher response to treatment compared to mesna-treated ones (Figure 1). The median effective dose (ED_50_) for iNOS, GSH, CAT, and SOD was 50 mg/kg.

### 2.2. Effect of Apigenin on the Expression of Pro-Inflammatory Cytokines Interleukin-6 (IL-6), Transforming Growth Factor 1-β (TGF 1-β), and Tissue Necrosis Factor-α (TNF-α)

**Figure 2:**
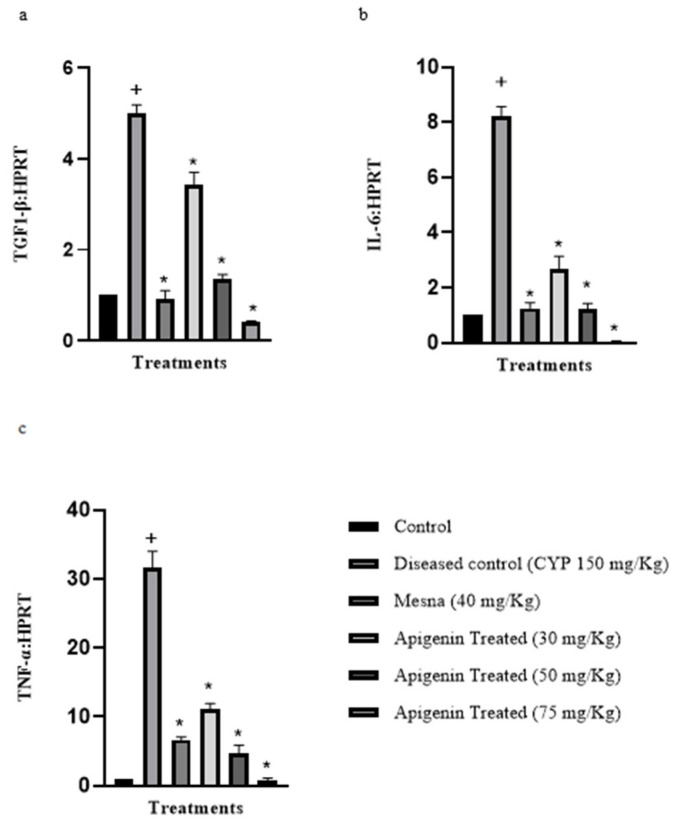
Reverse transcription polymerase chain reaction of cytokines. Relative gene expression of (**a**) interleukin-6 (IL-6), (**b**) transforming growth factor 1-β (TGF 1-β), (**c**) tissue necrosis factor -α (TNF-α), mRNA as normalized to Hypoxanthine-guanine phosphoribosyltransferase (HPRT) mRNA. The values were presented as mean ± standard error of mean *n* = 3. * *p* < 0.0001 relevant to the diseased control, ^+^ *p* < 0.0001 relevant to the control. One-way ANOVA followed by Tukey’s multiple comparison test was applied. SEM was represented by vertical bars.

The mRNA expression levels of pro-inflammatory cytokines (IL-6, TGF 1-β, and TNF-α) were evaluated. The levels were significantly high (*p* < 0.05) in CYP-treated subjects in comparison to the control. The subsequent doses of apigenin decreased the levels of the cytokines significantly (*p* < 0.05) in comparison to the diseased (Figure 2). The median effective dose (ED_50_) for IL-6, TGF 1-β, and TNF-α was 50 mg/kg.

### 2.3. Effect of Apigenin on Nociception

**Figure 3:**
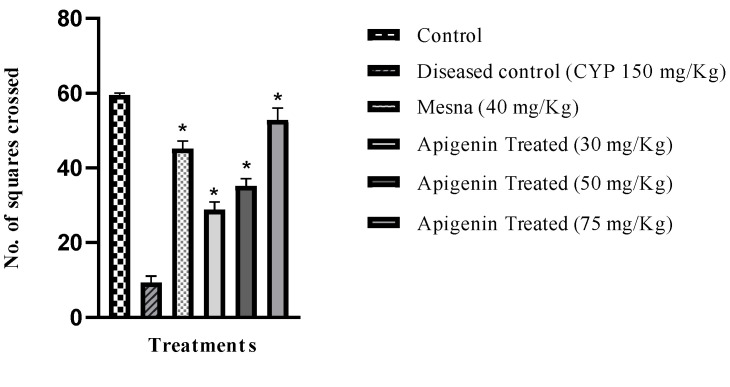
Effect of Apigenin on number of squares crossed by rats (locomotor index) in the control, the diseased control (CYP 150 mg/kg), and the mesna- (40 mg/kg) and apigenin-treated groups (30 mg/kg, 50 mg/kg, and 75 mg/kg). * *p* < 0.05 compared to the diseased control group. One-way ANOVA followed by Tukey’s multiple comparison test was applied, *n* = 6. SEM was represented by vertical bars.

For estimation of nociception, the locomotor index was measured by counting the number of box squares crossed by each rat for 10 min. The number of boxes crossed by CYP (150 mg/kg)-treated rats was significantly less compared to the control. These numbers were considerably improved in mesna (40 mg/kg) and apigenin (30 mg/kg, 50 mg/kg and 75 mg/kg) treated groups, respectively, as shown in (Figure 3). This test, however, had limitations.

### 2.4. Macroscopic Analysis

#### 2.4.1. Effect of Apigenin on Bladder Weight

The increase in bladder weight was a result of edema and hemorrhage. The CYP (150 mg/kg)-treated rats showed higher bladder weight compared to the control group. It was, however, observed that the rats treated with the mesna (40 mg/kg) and the apigenin (30 mg/kg, 50 mg/kg, and 75 mg/kg) showed reduction in bladder weight in a dose-dependent manner, as shown in Table 1.

#### 2.4.2. Effect of Apigenin on Hemorrhage and Edema

The CYP (150 mg/kg) treated rats exhibited marked edema and hemorrhage in comparison to the control rats. In the mesna- (40 mg/kg) and the apigenin-treated groups, these pathological features were, however, improved (Table 1).

### 2.5. Effect of Apigenin on Vascular Permeability

**Figure 4:**
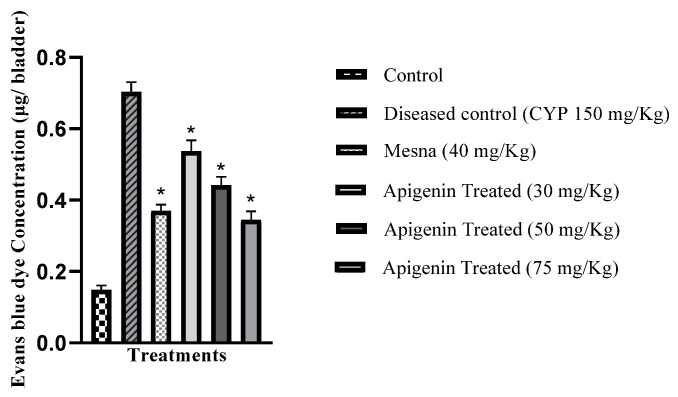
Effect of apigenin on vascular permeability of Evans blue dye in urinary bladder in the control, the diseased control (CYP 150 mg/kg), mesna- (40 mg/kg) and apigenin-treated groups (30 mg/kg, 50 mg/kg, and 75 mg/kg). * *p* < 0.05 compared to the diseased control group. One-way ANOVA followed by Tukey’s multiple comparison test was used, *n* = 6. SEM was represented by vertical bars.

The vascular protein leakage from vessels was estimated by Evans blue dye extravasation permeability. The extracted dye optical density was measured at 600 nm on a spectrophotometer. This study demonstrated increased Evans blue dye concentration in the diseased group that was significantly higher (*p* < 0.05) relative to the control group and decreased significantly (*p* < 0.05) in the apigenin-treated groups (Figure 4).

### 2.6. Histopathological Studies

**Figure 5:**
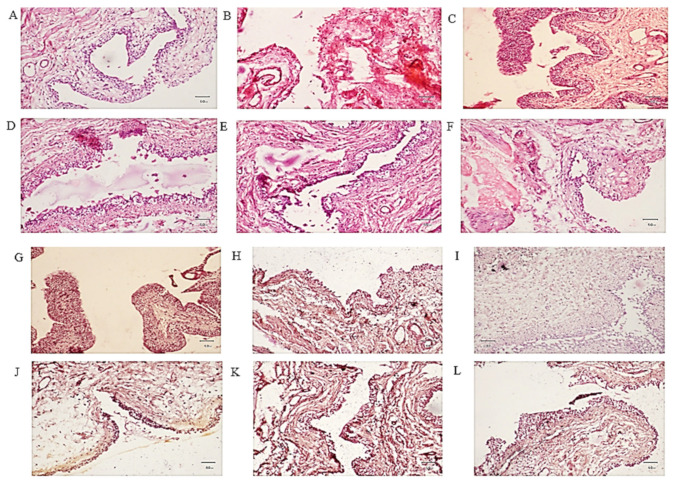
Hematoxylin and eosin (H and E) stain and periodic acid-Schiff (PAS) staining of female rat bladders. Bladders were fixed in 10% formalin, cryopreserved, cut serially into 5-µm sections, and stained. Hematoxylin and eosin (H and E) stain original magnification 10× of (**A**) the control showing normal bladder, (**B**) the cyclophosphamide (CYP)-treated group showing sloughing and degeneration of transitional epithelium, marked edema with inflammatory infiltrate and hemorrhage (**C**), the mesna (40 mg/kg) showing almost normal tissue, (**D**) apigenin (30 mg/kg body weight) to CYP post-treated rats showing marked proliferation of epithelial cells with hemorrhage, (**E**) apigenin (50 mg/kg body weight) to CYP post-treated rats showing mild sloughing and denudation of epithelium, and (**F**) the apigenin (75 mg/kg body weight) to CYP post-treated rats showing almost normal epithelium with no denudation. Periodic acid-Schiff stain of (**G**) the control showing normal urothelial GAG layer, (**H**) the cyclophosphamide (CYP)-treated rats showing urothelial damage with irregular GAG layer, (**I**) the mesna (40 mg/kg) showing mild urothelial damage with almost normal GAG layer, (**J**) apigenin (30 mg/kg body weight) to CYP post-treated rats showing marked proliferation of epithelial cells with damaged GAG layer, (**K**) the apigenin (50 mg/kg body weight) to CYP post-treated rats showing less GAG layer disturbance, and (**L**) the apigenin (75 mg/kg body weight) to CYP post-treated rats showing almost normal epithelium with no urothelial disturbance and GAG layer damage.

Isolated bladder tissues were analyzed for pathological changes. H and E staining was performed to rule out edema, hemorrhage, epithelial integrity, and lymphocytes infiltration, while periodic acid-Schiff staining was used to rule out glycosaminoglycan. Bladders of the control animals showed normal bladder tissue with no inflammation, hemorrhage, or edema (Figure 5A), while high levels of epithelial denudation, hemorrhage, and edema in the diseased rats (Figure 5B) were observed. Pretreatment with apigenin prevented tissue architecture damage of the bladder in H and E staining, as shown in (Figure 5D–F). Pretreatment with the standard drug, mesna, showed the same set of results (Figure 5C). PAS staining was performed to check urothelium and GAG layer integrity. The control showed intact GAG layers (Figure 5G). Urothelium and GAG layer were severely damaged in the CYP-treated group (Figure 5H). The mesna-pretreated group showed mild urothelial damage with almost normal GAG layers (Figure 5I). The apigenin pretreatment in increasing doses restored the urothelium and GAG layer (Figure 5J–L).

### 2.7. Molecular Docking

**Figure 6:**
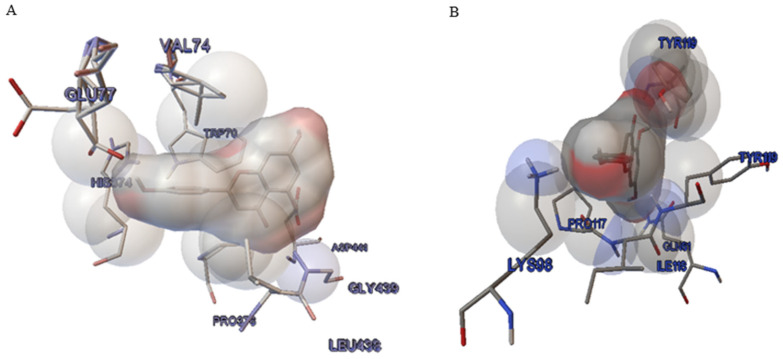
(**A**) Ligand (api/shown in white sphere) interaction with amino acid residues (shown as sticks) of 3DK4 enzyme. (**B**) Amino acid residues (shown as sticks) of 4TWT enzyme with best docked structure of ligand api (shown in gray-red sphere) modeled by Auto Dock Tools.

The three-dimensional coordinates of apigenin and mesna were retrieved from PubChem with their respective PubChem CIDs. The affinities of ligands with various antioxidant enzymes and pro-inflammatory cytokines were tabularized in Table 2. The mesna served as the standard for entire receptors. In Homo sapiens, the 3D modeling of all receptors was projected through Auto Dock Tools 1.5.6; developed by Scripps Research in 1989 under GNU General Public Licence (https://autodock.scripps.edu, accessed on 1 April 2023). Auto Dock created 10 clusters, and the cluster with the minimum binding energy was considered suitable for pharmacological evaluation. Organizational evaluations and validation of the protein model were performed by Pyrex. In order to obtain optimal alignment, the path with the best RMSD was subjected to dynamic programming. As it was cleared from the table, the apigenin showed good interactions at CAT, GSH, IL-6, and TNFα receptors in comparison to the standard drug mesna, which demonstrated poor interactions at similar receptors. The apigenin demonstrated the best interaction at the GSH receptor (3DK4) for antioxidant activity, along with the least binding energy of −9.44 Kcal/mol. The main interacting residues are TRP70, VAL74, GLU77, PRO373, HIS374, LEU438, GLY439, and ASP441 with two hydrogen bonds, as shown in Figure 6A. The best docked conformation of apigenin at the 1TPO receptor represented one hydrogen bond. However, it also shows best activity at the TNFα (4TWT) receptor, presenting the least binding energy of −8.77 Kcal/mol. As shown in Figure 6B, the main interacting residues of 4TWT were GLN61, LYS98, ILE116, PRO117, and TYR119 with one hydrogen bond.

### 2.8. Effect of Apigenin on Isolated Rat Bladder Strips

#### 2.8.1. Carbachol Contraction Response in Isolated Bladder Strips

**Figure 7:**
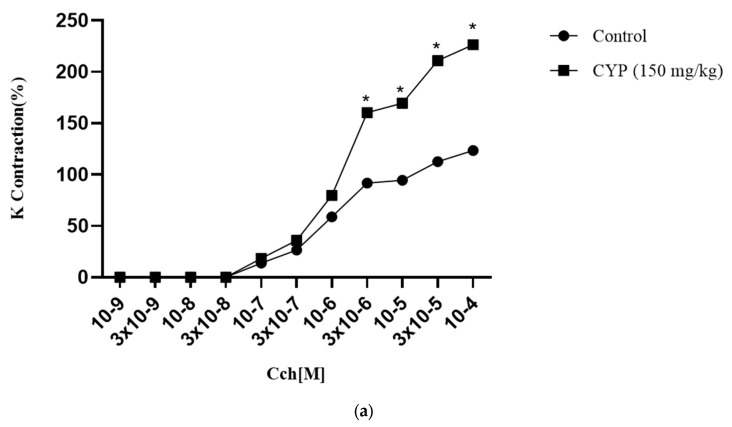

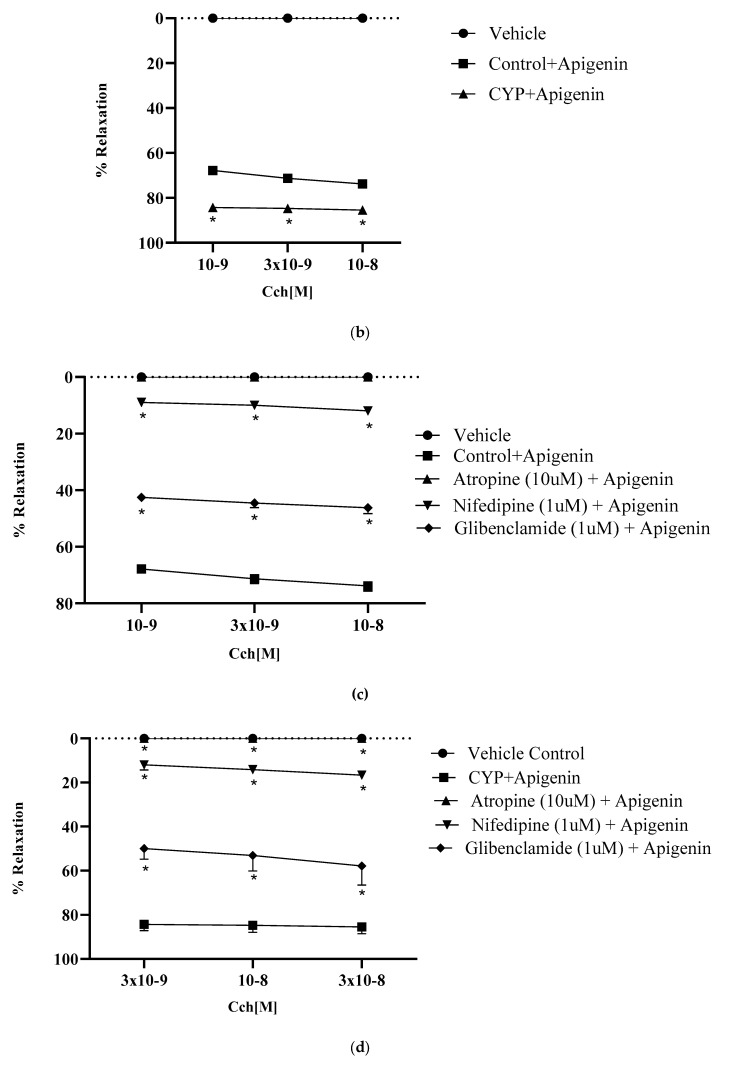

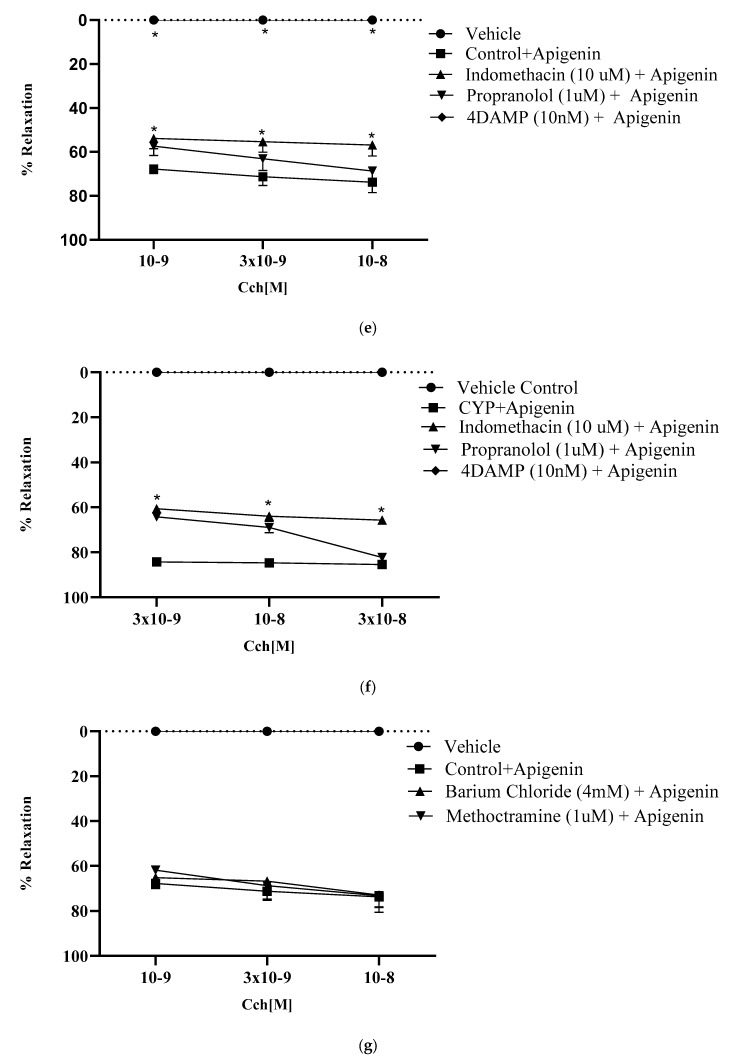

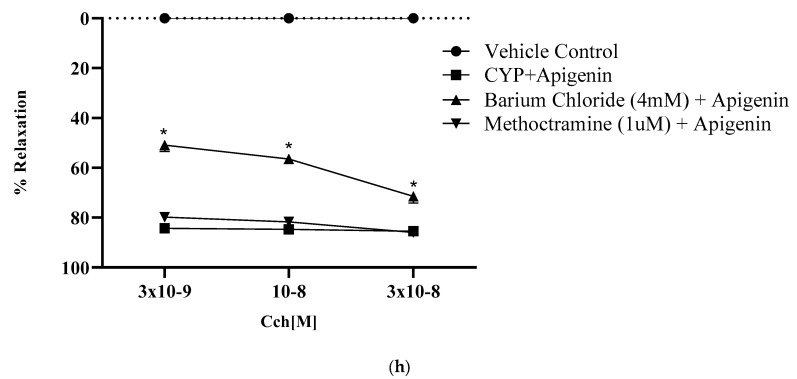
(**a**) The cumulative carbachol (Cch) dose response curves. In isolated rat bladder strips, carbachol responses (10^−9^ to 10^−4^) from the control and cyclophosphamide-treated rats. Data have been presented as mean ± SEM. Two-way ANOVA followed by Bonferroni’s multiple comparison test was applied, *n* = 6, * *p* < 0.05, significant compared to the control. (**b**) The cumulative apigenin response curves. In isolated rat bladder strips, cumulative apigenin (10^−9^ to 10^−8^) responses from the control, the vehicle control, and the cyclophosphamide-treated rats. Data have been presented as mean ± SEM. Two-way ANOVA followed by Bonferroni’s multiple comparison test was applied, *n* = 6, * *p* < 0.05, significant compared to the control. (**c**) The cumulative apigenin response curves alone and after pre-incubation with antagonists. In isolated rat bladder strips, the cumulative apigenin response (10^−9^ to 10^−8^) alone and in the presence of atropine (10 μM), nifedipine (1 μM), and glibenclamide (1 μM). Data have been presented as mean ± SEM. Two-way ANOVA followed by Tukey’s multiple comparison test was applied, *n* = 6, * *p* < 0.05, significant compared to the group treated with apigenin alone. (**d**) The cumulative apigenin response curves in cyclophosphamide-treated rats alone and after pre-incubation with antagonist. In isolated rat bladder strips, the cumulative apigenin response (10^−9^ to 10^−8^) in cyclophosphamide-treated isolated rat bladder strips alone and in the presence of atropine (10 μM), nifedipine (1μM), and glibenclamide (1μM). Data have been presented as mean ± SEM. Two-way ANOVA followed by Tukey’s multiple comparison test was applied, *n* = 6, * *p* < 0.05, significant compared to the group treated with apigenin alone. (**e**) The cumulative apigenin response curves alone and after pre-incubation with antagonist. In isolated rat bladder strips, the cumulative apigenin response (10^−9^ to 10^−8^M) alone and in the presence of indomethacin (10 μM), propranolol (1 μM), and 4DAMP (10 nM). Data have been presented as mean ± SEM. Two-way ANOVA followed by Tukey’s multiple comparison test was applied, *n* = 6, * *p* < 0.05, significant compared to the group treated with apigenin alone. (**f**) The cumulative apigenin response curves in cyclophosphamide-treated rats alone and after pre-incubation with antagonist. In isolated rat bladder strips, the cumulative apigenin response (10^−9^ to 10^−8^M) in cyclophosphamide-treated isolated rat bladder strips alone and in the presence of indomethacin (10 μM), propranolol (1 μM), and 4 DAMP (10 nM). Data have been presented as mean ± SEM. Two-way ANOVA followed by Tukey’s multiple comparison test was applied, *n* = 6, * *p* < 0.05 significant compared to the group treated with apigenin alone. (**g**) The cumulative apigenin response curves alone and after pre-incubation with antagonist. In isolated rat bladder strips, the cumulative apigenin response (10^−9^ to 10^−8^M) alone and in the presence of barium chloride (4 mM) and methoctramine (1 μM). Data have been presented as mean ± SEM. Two-way ANOVA followed by Tukey’s multiple comparison test was applied, *n* = 6, * *p* < 0.05, significant compared to the group treated with apigenin alone. (**h**) The cumulative apigenin response curves in cyclophosphamide-treated rats alone and after pre-incubation with antagonist. In isolated rat bladder strips, the cumulative apigenin response (10^−9^ to 10^−8^M) in cyclophosphamide-treated isolated rat bladder strips alone and in the presence of barium chloride (4 nM) and methoctramine (1 μM). Data have been presented as mean ± SEM. Two-way ANOVA followed by Tukey’s multiple comparison test was applied, *n* = 6, * *p* < 0.05, significant compared to the group treated with apigenin alone.

Carbachol contractions were expressed as percentage response to 80 mM KCl. The contraction obtained with 80 mM KCl in the control and diseased groups remained non-significant, whereas carbachol-induced contractions were elevated (226 ± 2.05%) significantly (*p* < 0.05) in the CYP-treated animals compared to the control (123 ± 1.3%) (Figure 7a).

#### 2.8.2. Relaxant Response of Apigenin against Carbachol-Induced Contractions

Apigenin (10^−9^ to 10^−8^) produced a relaxant response against carbachol-induced contractions in both control and diseased rats. However, the response was more significant (*p* < 0.05) in the diseased rats than in the control (Figure 7b). There was a significant (*p* < 0.05) difference in EC_50_ values of the control (9.578 × 10^−11^ ± 1M) and the CYP-treated rats (1.336 × 10^−11^ ± 1M).

#### 2.8.3. Effect of Muscarinic Receptors on Relaxant Potential of Apigenin

To determine the role of muscarinic receptors in the apigenin-induced relaxant effect, the non-selective muscarinic receptor blocker atropine (10 μM), the M_3_ selective blocker 4DAMP (10 nM), and the M_2_ selective blocker methoctramine (1 μM) were used during experimentation. The relaxant response of apigenin was completely abolished by atropine and 4DAMP in the control and the diseased (Figure 7c–f), while no change in response was observed with methoctramine incubated and non-incubated strips (Figure 5G,H). The EC_50_ values of methoctramine pre-incubated control strips (2.186 × 10^−10^ ± 1M) was higher in relation to the diseased (8.840 × 10^−11^ ± 1M).

#### 2.8.4. Effect of Nifedipine on Relaxant Potential of Apigenin

The spasmolytic effect of the apigenin was decreased significantly (*p* < 0.05) in the control (12 ± 0.8%) and the diseased (16 ± 0.6%) groups when incubated with nifedipine (1 μM), a calcium channel blocker (Figure 7c,d). The EC_50_ values of nifedipine pre-incubated control strips (3.746 × 10^−10^± 1M) were low in comparison to the diseased (4.425 × 10^−10^ ± 1 M).

#### 2.8.5. Role of K_ATP_ Channel Blocker in Relaxant Effect of Apigenin

Glibenclamide (1 μM), a K_ATP_ channel blocker, reduced relaxation produced by apigenin significantly (*p* < 0.05) to 46 ± 2.1% in the control and 57.8 ± 3.6% in the cyclophosphamide-treated groups (Figure 5C,D). The EC_50_ values of strips pre-incubated with glibenclamide in the diseased (1.481 × 10^−10^ ± 1M) were greater than those in the control (1.011 × 10^−10^ ± 1 M).

#### 2.8.6. Role of K_IR_ Channels in Relaxant Effect of Apigenin

With an inward rectifier potassium channel blocker, barium chloride (4 mM), no difference in relaxation was observed in the control, while a slight decrease in relaxation in response to the apigenin was observed in the diseased (Figure 7g,h). The EC_50_ values of barium chloride pre-incubated strips in the diseased (4.914 × 10^−10^ ± 1 M) were greater in comparison to the control (1.192 × 10^−10^ ± 1 M).

#### 2.8.7. Involvement of β- Adrenergic Receptors in Relaxant Effect of Apigenin

Propranolol (1 μM), a non-selective β-adrenergic receptor blocker, non-significantly lowered the relaxation effect of apigenin in the diseased (82 ± 1.8%) while declining it significantly (*p* < 0.05) in the control (68 ± 2.2%) (Figure 7e,f). There was a difference in the EC_50_ values of strips pre-incubated with propranolol. The values were higher in the diseased (3.064 × 10^−10^ ± 1 M) than in the control (2.128 × 10^−10^ ± 1 M).

#### 2.8.8. Role of Prostaglandins in Relaxant Effect of Apigenin

A prostaglandin synthesis inhibitor, indomethacin (10 μM), attenuated the relaxant effect of apigenin significantly to 56.8 ± 2.0% in the control and 65 ± 1.9% in the diseased (Figure 7e,f). In strips pre-incubated with indomethacin, the EC_50_ values in the diseased (9.520 × 10^−11^ ± 1 M) were higher than in the control (6.222 × 10^−11^ ± 1 M).

## 3. Discussion

Interstitial cystitis is a chronic inflammation of the bladder. It is often accompanied by severe pain, increasing micturition, and increased voiding frequency [21]. Repeated CYP injections of 150 mg/kg every third day for a total of seven days induces inflammation, submucosal edema, lymphocyte infiltration, and hemorrhage, along with sloughing and denudation of the epithelium [22]. Acrolein, a metabolite of CYP, is responsible for the pathophysiological aspects of the disease. It concentrates in the urothelium due to its chemical nature, where it increases production of ROS (reactive oxidative species). ROS intensifies the expression of various transcription factors, like interleukins, tissue necrosis factor-α (TNF-α), transforming growth factor 1-β (TGF 1-β), and inducible nitric oxide synthase (iNOS), while decreasing antioxidants such as superoxide dismutase (SOD) and catalase (CAT) [5,6]. Bladder muscles are hyperexcited, with increasing micturition frequency and decreasing urine-retaining capacity [14,23]. The current study was aimed to identify the beneficial role of apigenin for treatment of CYP-induced interstitial cystitis. Apigenin is a plant flavonoid found in various fruits, vegetables, and medicinal plants, such as chamomile and oranges. An apigenin-rich diet has anti-inflammatory, antioxidant, and immune system regulatory roles [24].

The SOD, CAT, and GSH were the main enzymes of cellular defense that were used as an indicator of inflammation. The reduced expression of these enzymes points to establishment of disease. This study demonstrated that the expression of these antioxidants’ enzymes decreased in the CYP treatment. SOD and CAT were vital in maintaining redox status of cells. SOD catalyzes superoxide (O_2_^−^) into oxygen and hydrogen peroxide (H_2_O_2_), whereas CAT further degrades H_2_O_2_ into O_2_ and H_2_O. ROS was a key culprit of urothelial barrier damage. It was produced by inflammatory cells upon CYP insult [12,23]. Apigenin pretreatment was shown to improve levels, owing to the antioxidant potential. It produced a protective response in CYP-induced interstitial cystitis by improving levels of SOD and CAT. GSH is a thiol antioxidant that protects cells against oxidants. It also plays an important role in redox reactions. Depletion of GSH has been reported in a variety of inflammatory diseases [25]. Apigenin pretreatment increased declined level of GSH in comparison to the CYP-treated group. Decreased cellular defense was further manifested by generation of ROS and RNS (reactive nitrogen species) as a result of acrolein accumulation. The iNOS levels tend to be high in CYP-injected bladder tissues; apigenin lowered the raised levels significantly [5]. Apigenin may be beneficial in CYP-induced IC through decreasing generation of RNS. ROS and RNS also enhance expression of pro-inflammatory cytokines like IL-6, TNF-α, and TGF 1-β related to CYP administration. The expression of these pro-inflammatory cytokines was raised in the CYP-treated group [5,24]. Apigenin significantly decreased the level of these cytokines in a dose-dependent manner. Hence, apigenin demonstrated antioxidant and anti-inflammatory activity.

Pain is a symptom of inflammation. Animals express pain with certain specific responses, like crying, licking, piloerection, intra-abdominal contractions, and abnormal facial expressions. Researchers use locomotor activity to analyze inflammatory pain [26]. Locomotor activity was significantly suppressed in the CYP-treated group in comparison to the control. Apigenin pretreatment (30 mg/kg, 50 mg/kg, and 75 mg/kg) significantly restored locomotor activity. The pain symptoms in animals were abolished with apigenin pretreatment. Apigenin presented marked analgesic activity. These results were consistent with previous studies. The limitation of this study was non- specificity [27].

Edema and hemorrhage were due to inflammation that destroyed the urothelial barrier. Gray’s criteria were adopted to express edema and hemorrhage. The CYP-treated group showed marked edema and hemorrhage, while apigenin pretreatment prevented tissue damage of the bladder. Bladder wet weight indicates edema in the bladder. It was profoundly raised in the CYP-treated group, while apigenin pretreatment (30, 50, and 75 mg/kg) significantly decreased edema. Evans blue dye had an affinity for serum albumin. This dye extravasation showed capillary leakage as a sign of edema following inflammation. Both mesna (40 mg/kg) and apigenin (30, 50 and 75 mg/kg) pretreatment decreased Evans blue leakage compared to the CYP-treated group. Anti-inflammatory substances that inhibited tissue damage showed the same set of responses as in previous studies [28]. Histological evaluation indicates bladder tissue damage. The control group revealed normal epithelia and GAG layer. CYP treatment induced marked hemorrhage, edema, and denudation of epithelium with damaged GAG layer. Pretreatment with mesna (40 mg/kg) and apigenin (30 mg/kg, 50 mg/kg, and 75 mg/kg) inhibited bladder tissue damage. Anti-inflammatory substances showed the same responses in earlier studies [29].

The in silico method was used in the drug discovery process. It explains the interaction of a ligand with its relevant target [30]. Computational in silico studies were conducted to display phytochemical antioxidant potential against enzymes SOD, CAT, and GSH [31]. This study revealed that apigenin interacts better with antioxidant enzymes. It inhibited both pro-inflammatory cytokines and oxidant enzymes. Early reports suggested that the compounds presenting inhibitory potential with iNOS, TNF-α, IL-6, and TGF1-β had decent anti-inflammatory activity [32,33].

A series of experiments were conducted on isolated rat bladder strips from control and diseased rats to determine the spasmolytic effect of apigenin. To explain the possible mechanisms involved in the relaxant effect of apigenin, the strips were pre-incubated with several receptors and channel blockers individually.

In the bladder, all subtypes of muscarinic receptors (M_1_–M_5_) are found in relative abundance. Bladder epithelium and detrusor smooth muscle have muscarinic M_2_ and M_3_ subtypes. Their population, however, is changed in pathological conditions like IC [34]. In the present study, the carbachol-induced contractile response was significantly higher in the diseased group compared to the control group, while contraction with 80 mM KCl remained the same in both groups, as reported in earlier research [14]. Apigenin demonstrated a spasmolytic effect in control and diseased bladder strips. Greater relaxation was observed in the CYP-treated group compared to the control.

Muscarinic receptor antagonists are used in overactive bladder disease. The carbachol-induced contractile response was completely eradicated by apigenin indiscriminately in both control and diseased rats. The results emphasized apigenin’s ability to inhibit M_3_ receptors. M_2_ receptors were not related to the relaxation induced by apigenin, as the relaxation remained the same in methoctramine (1 μM) incubated and non- incubated strips [35]. The K_ATP_ channel blocker possessed a relaxant response. K_ATP_ channels are found abundantly in detrusor smooth muscles. Glibenclamide is a sulphonyl urea that blocks voltage-insensitive K_ATP_ channels [36]. Glibenclamide (1 μM) was added to a bath. In the diseased group, high relaxation was detected compared to the control, indicating the role of these channels in apigenin relaxation. K_IR_ channels were unrelated to relaxation of apigenin in the control, whereas slight relaxation was shown in the diseased with barium chloride (4 mM) [37].

L-type calcium channels have been identified in the bladders of guinea pigs and humans. These channels show sensitivity to nifedipine-inhibited contraction of detrusor via decreasing extracellular calcium flux [38]. The relaxant effect of apigenin was decreased significantly in the diseased and control groups when pre-incubated with nifedipine (1 μM), pointing to calcium channel blocking as one of the mechanisms involved in relaxation induced by apigenin [39]

Beta adrenergic receptors are present in the bladder. β- receptors may be traced in the urothelium, where they change the detrusor function by modulating the release of various factors [40]. The strips were pre-incubated with propranolol (1 μM), a nonselective β adrenergic receptor blocker. It did not reduce relaxation induced by apigenin, indicating that β adrenergic receptors are not involved in relaxation caused by apigenin.

Prostaglandin (PG) subtypes are present in the entire urinary tract. PG E and F are in detrusor muscles, leading to contraction. The involvement of PG was assessed by giving indomethacin (10 μM). The relaxant effect was decreased significantly in the control and the diseased. It may be one of the possible mechanisms of relaxation, as reported in earlier studies [41].

## 4. Materials and Methods

### 4.1. Chemicals Used

Only analytical grade chemicals were used in the study. These comprise cyclophosphamide, mesna, atropine, glibenclamide, indomethacin, propranolol, 4-diphenylacetoxy-N-methylpiperidine (4 DAMP), methoctramine, barium chloride, nifedipine, carbachol, potassium dihydrogen phosphate (KH_2_PO_4_), potassium chloride (KCl), sodium chloride (NaCl), sodium bicarbonate (NaHCO_3_), calcium chloride (CaCl_2_), glucose monohydrate (C_6_H_12_O_6_. H_2_O), and magnesium sulphate (MgSO_4_). Both mesna and cyclophosphamide (Sigma-Aldrich) were purchased from the Department of Oncology, Sheikh Zaid Hospital Lahore, Lahore. Sterile normal saline (0.9%) was added to CYP to make an injectable solution. Apigenin (99%) was purchased from Sigma-Aldrich (CAS 520-36-5) and was dissolved in 0.5% carboxymethylcellulose sodium (CMC Na).

### 4.2. Animals Used

All the experiments were conducted on adult female rats (Sprague−Dawley) weighing 250 ± 10 g, purchased from the Pharmacology Department of the University of Lahore, Lahore. All the animals were housed in relative humidity (60 ± 5%), temperature (21 ± 3 °C), and light (12 light/dark cycles), with free accessibility to water ad libitum and standard chow food. All the animals were acclimatized for the duration of a week before commencing the study. All experimental procedures were approved and monetized by the Institutional Research and Ethical Committee of the Faculty of Pharmacy (IREC-2022-05), the University of Lahore, Lahore, Pakistan.

### 4.3. Cyclophosphamide-Induced Interstitial Cystitis

**Figure 8:**
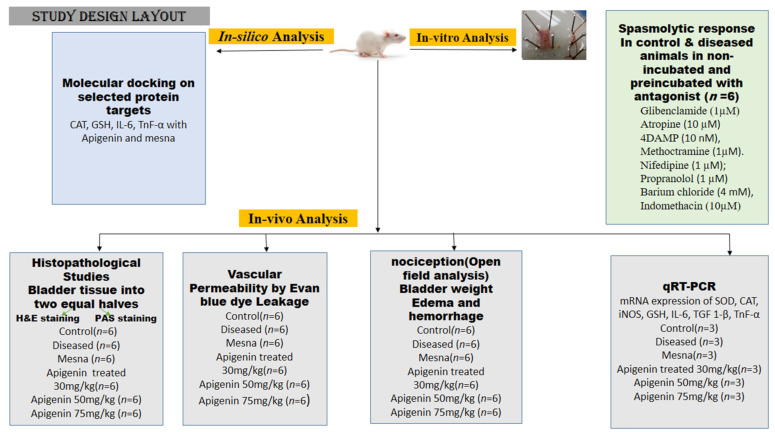
A schematic representation for estimation of uroprotective and spasmolytic response of apigenin; in-vivo, in-vitro, and in-silico analysis.

Upon acclimation to laboratory conditions, the female rats were injected with CYP 150 mg/kg body weight on days 1, 4, and 7. On day 8, animals were sacrificed to be used in the study as shown in Figure 8 [5].

### 4.4. Study Design

All the rats were randomly divided into six groups (*n* = 6). The control received normal saline (0.9 % NaCl) on days 1, 1, and 7 through the intraperitoneal route (IP). The diseased control received CYP (150 mg/kg) that was injected through the intraperitoneal route on days 1, 4, and 7. In the mesna-treated group, mesna (40 mg/kg) was administered orally 1 h ahead of CYP injection on days 1, 4, and 7. In the apigenin-treated group, apigenin (30 mg/kg, 50 mg/kg, and 75 mg/kg) was administered orally 1 h prior to CYP IP injection on days 1, 4, and 7, respectively as shown in Figure 8.

### 4.5. Quantitative Real-Time PCR Analysis for Measuring mRNA Expression of SOD, CAT, iNOS, GSH, IL-6, TGF 1-β, TNF-α

On day 8, bladder tissue from each rat was dissected out to be used in the study. From cells, entire RNA was isolated with the help of TRIzol (TRI) reagent (Sigma Chemical Company). The complementary DNA (cDNA) was synthesized with the WizScript cDNA Synthesis Kit (Wizbio Solutions, New Mexico, USA). To do so, SYBR Green qPCR mix (Zokeyo, Wuhan, China) was utilized. To measure mRNA, the following conditions were observed during experimentation: denaturation initially for 2 min at 94 °C following denaturation for 40 cycles at 94 °C for 1 min then final annealing at 60 °C for 2 min. The 2^−∆∆CT^ method was used to express comparative transcription of various genes with Hypoxanthine-guanine phosphoribosyltransferase (HRPT) as an internal reference gene Figure 8 [42].

### 4.6. Assessment of Nociception

Prior to behavioral testing, the rats were positioned independently in observation boxes and kept in them for half an hour. The behavioral changes to be evaluated were (1) related to locomotion such as walking, climbing, grooming, etc.; (2) related to immobility; and (3) related to pain, such as cries. The parameters were coded as 0 for normal; 1 for hair bristling; 2 for extensive hair bristling; 3 for problems in breathing; 4 for licking of stomach; and 5 for abdominal extension and shrinkage. To record nociception in animals, all rats were kept under observation for 120 s. A session was held each half hour for a total of 4 h after injecting CYP. The results were summarized at the end of an hour-long observation session. To measure the locomotor activity index, the rats were put in a container separated into nine squares. The number of squares crossed by each rat with four paws was then counted. This represented the locomotor index [5].

### 4.7. Assessment of Bladder Edema and Hemorrhage

Rats were executed to collect the bladders. The bladders of all the animals were individually weighed. Increase in the bladder wet weight is due to the presence of edema expressed in mg. Gray’s criteria were adopted for the superficial examination of edema and hemorrhage in urinary bladders. The animal bladders were visualized. According to Gray, acute edema was represented as (3+) severe, (2+) moderate, (1+) mild, or (0) no edema. When fluid was evident in both the external and internal portions of the bladder walls, the edema was categorized as severe. When the fluid retention was limited to the inner mucosa, it was characterized as moderate; the least fluid retention was considered mild edema. For the hemorrhage score, the criteria used (3+) for intravesical clots, (2+) for mucosal hematomas, (1+) for blood vessel dilatation, and (0) for normal morphology Figure 8 [5].

### 4.8. Evaluation of Vascular Protein Leakage by the Evans Blue Dye Technique

The rats were given an intraperitoneal injection of cyclophosphamide along with an infusion of Evans blue dye (25 mg/kg) through the intravenous route half an hour before killing. After the killing of the rats, bladders were removed, divided, and cultivated for extraction of dye in an incubator at 56 °C for 6 h or overnight in tubes containing formamide solution (1 mL/bladder). The concentration of extracted dye was calculated by measuring absorbance at 600 nm and equating it to the standard curve of Evans blue dye µg/bladder [16].

### 4.9. Histologic Analysis

Upon completion of dosing, the bladders of the animals were obtained to perform further histopathological analysis. Each bladder was cut into two equal halves. Each section was preserved in a 10% formalin solution. The tissues were stained with hematoxylin–eosin (H and E) and periodic acid-Schiff (PAS) staining for the urothelium glycosamine glycan (GAG) layer with a routine protocol. The H and E-stained section was examined for edema, leukocyte infiltration, and urothelium damage while PAS stained for glycosaminoglycan as presented in Figure 8 [1,2].

### 4.10. Molecular Docking

#### 4.10.1. Ligand Selection

To search for new anti-inflammatory substances, a coordination sphere of ligands was prepared. The apigenin (Api) two-dimensional structure (https://pubchem.ncbi.nlm.nih.gov/compound/5280443 (accessed on 20 May 2023) and mesna (https://pubchem.ncbi.nlm.nih.gov/compound/29769 accessed on (24 June 2005) were acquired from the Pub Chem database in SDF file format and changed to PDB format by Open Babel GUI 3.0 software. Open babel development team release this software in 2005 with GNU general public license [43]. Afterwards, the PDB file of ligands was used for programmed docking at several receptors through Auto Dock Tools software [44].

#### 4.10.2. Characterization of Receptor

The crystalline assembly of catalase (CAT), glutathione (GSH), and cytokines (IL-6, TNFα) was obtained from the RCSB Protein Data Bank (PDB) with PDB ID: 1DGB (https://www.rcsb.org/structure/1dgb (accessed on 11 February 2000)) for the antioxidant receptor (CAT); PDB ID: 3DK4 (https://www.rcsb.org/structure/3dk4 (accessed on (5 August 2008) for the antioxidant receptor (GSH); PDB ID: 4ZS7 (https://www.rcsb.org/structure/4zs7 (accessed on 4 May 2016) for the interleukin 6 receptor (IL-6) for pro-inflammatory activity, and PDB ID: 4TWT (https://www.rcsb.org/structure/4twt) (accessed on 1 April 2023) for the tumor necrosis factor (TNF) for estimation of pro-inflammatory potential. The crystalline assembly of receptors was exhibited through Auto Dock Tools 1.5.6 purified, whereas polar hydrogen atoms and partial charges were incorporated. To demonstrate ligand interaction macromolecule was saved in its PDBQT format [45]. The preeminent active region of enzymes with amino acid residues was highlighted by pointing to the binding site utilized in binding to the ligand [46].

#### 4.10.3. Docking Studies

The protein ligand interactions were predicted by docking studies. Here, mesna (mes) served as the standard drug at various receptors for antioxidant and anti-inflammatory studies. Receptor-ligand interaction insight was possible with a computational drug design platform [47]. Scoring and molecular docking of numerous receptors were calculated by Auto Dock Tools 1.5.6 software; it mimics the binding conformations of ligands to examine the binding sites of docked molecules and uses the Lamarckian genetic algorithm (LGA). The grid box was set at 70 × 70 × 70 Å along the X, Y, and Z axes with the Auto Dock requirements as follows: 1. Genetic algorithm with populace size = 150; 2. Extreme quantity of energy estimations = 2,500,000; 3. Genetic algorithm cross-over type = 2 points. The parameters of rigidity were established for the receptor ensuring the flexibility of the ligand. The chemical and configurational properties of the active site permit identification and binding of ligands. Subsequently, protein-ligand docking at diverse receptors gave ten docked conformations (poses); the finest conformation was assessed in terms of least binding energy between numerous binding communications. Cluster examination of protein binding sites with least binding energy was further conducted by utilizing Pymol Molecular Graphics System offline software [44].

#### 4.10.4. Model Validation

The re-docking method was utilized to validate the docking program; in both scenarios within the active site of the enzyme, the co-crystallizing ligands were redocked. Native ligand interactions along with RMSD inside the crystalline protein structure were considered as standard docked models. To forecast the binding orientation of the ligand root, mean square deviation (RMSD) was calculated and <2 Å was kept as accurate [44].

### 4.11. In-Vitro Activity on Isolated Rat Bladder Strips

Upon the termination of the rats, the bladders of the control and CYP-treated groups were surgically excised. They were cut longitudinally to get four strips from each bladder. The bladder was submerged in 20 mL warm aerated Krebs-Henseleit solution. The tissue was equilibrated for 1 h at 1 g baseline tension. The tissue’s viability was checked after adding KCl (80 mM). Contractile response was detected by adding cumulative carbachol doses from 10-9 M to 10-4 M. The apigenin relaxant effect was measured against carbachol-induced contractions. To access the role of relevant receptors involved in relaxation, the strips were pre-incubated with indomethacin (10 μM), propranolol (1 μM), 4DAMP (10 nM), atropine (10 μM), nifedipine (1 μM), glibenclamide (1 μM), barium chloride (4 mM), and methoctramine (1 μM) half an hour prior to carbachol application presented in Figure 8. LabChart 8 power lab data acquisition system (AD instruments; USA) connected to a computer Power Lab University of Lahore, was used to analyze tissue contraction [48,49].

### 4.12. Statistical Analysis

The data have been presented as mean ± SEM. The data obtained from several groups were analyzed statistically by one-way/two-way analysis of variance (ANOVA) following Tukey’s/Bonferroni’s multiple comparison tests by means of Graph Pad Prism version 8.0.2 depending on type of analysis. *p* ≤ 0.05 was considered statistically significant [5].

## 5. Conclusions

The current study demonstrated that apigenin possesses spasmolytic and uroprotective effects against interstitial cystitis. At all experimental concentrations, it ameliorated interstitial cystitis symptoms by reducing bladder weight, vascular permeability, and expression levels of IL-6, TNF-α, TGF 1-β, and iNOS, while increasing SOD, CAT, and GSH levels. In silico analyses also confirmed the antagonizing potential of apigenin against these mediators. Apigenin induced a relaxant effect on bladder smooth muscles. It produces relaxation against carbachol-induced contractions, possibly by blocking M_3_ receptors, K_ATP_ channels, L-type calcium channels, and prostaglandin inhibition. Thus, apigenin might be a potential agent in the treatment of interstitial cystitis.

## Figures and Tables

**Table 1:** Effect of apigenin on bladder wight, edema, and hemorrhage.

Groups	Bladder Weight (mg) Mean ± SEM	Nociception Mean ± SEM	Edema Mean ± SEM	Hemorrhage Mean ± SEM
Control	9.0 ± 0.36 *	0 ± 0.001 *	0 ± 0.01 *	0 ± 0.001 *
Diseased control (CYP 150 mg/kg)	17.16 ± 0.40	1, 2, 3, 4, 5 ± 0.01	3+ ± 0.001	3+ ± 0.01
Mesna (40 mg/kg)	11.40 ± 0.51 *	1 ± 0.001 *	1+ ± 0.01 *	0 ± 0.001 *
Apigenin Treated (30 mg/kg)	13.00 ± 0.36 *	1, 2 ± 0.02 *	2+ ± 0.02 *	3+ ± 0.01 *
Apigenin Treated (50 mg/kg)	10.50 ± 0.34 *	1 ± 0.04 *	1+ ± 0.02 *	2+ ± 0.02 *
Apigenin Treated (75 mg/kg)	9.16 ± 0.30 *	0 ± 0.01 *	1+ ± 0.01 *	1+ ± 0.02 *

*p* * < 0.001 significant compared to the diseased control group.

**Table 2:** Ligands apigenin and mesna interactions with various antioxidant, anti-inflammatory, and anticancer receptors.

Receptor	Active Compound	PDB ID	Binding Energy (Kcal/mol)	Inhibition Constant (ki) (nM)	Torsional Energy	No of Hydrogen Bonds	Interacting Residues
Catalase-Apigenin	CAT(antioxidant)	1DGB	−6.9	8.28	1.19	2	THR239, PRO241, LEU245, TYR246, ASP238, TYR246
Glutathione- Apigenin	GSH(antioxidant)	3DK4	−9.44	121.2	1.19	2	TRP70, VAL74, GLU77, PRO373, HIS374, LEU438, GLY439, ASP441
Cytokine- Apigenin	IL-6 (pro-inflammatory)	4ZS7	−7.49	3.25	1.19	1	VAL2, GLN3, LEU4, ARG99, VAL24, TYR111, TRP112, GLN114
Tumor necrosis factor- Apigenin	TNF-α (pro-inflammatory)	4TWT	−8.77	375.5	1.19	1	GLN61, LYS98, ILE116, PRO117, TYR119
Catalase-Mesna	CAT (antioxidant)	1DGB	−4.51	496.9	0.3	1	LEU26, PRO78, THR79, LEU80, VAL132, LEU134
Glutathione- Mesna	GSH (antioxidant)	3DK4	−5.01	210.95	0.3	2	GLY29, SER30, GLY56, THR57, CYS58, THR339
Cytokine- Mesna	IL-6 (pro-inflammatory)	4ZS7	−4.87	270.44	0.3	1	LEU47, GLU48, TRP49, ARG94, ALA99, VAL100, PHE101S
Tumor necrosis factor- Mesna	TNF-α (pro-inflammatory)	4TWT	−4.67	379.61	0.3	2	VAL17, PHE144, GLU146, GLY148, GLN149, VAL150, TYR151

## Data Availability

Data is contained within the article.

## References

[1] Smaldone M.C., Vodovotz Y., Tyagi V., Barclay D., Philips B.J., Yoshimura N., Tyagi P. (2009). Multiplex analysis of urinary cytokine levels in rat model of cyclophosphamide-induced cystitis. Urology.

[2] Chung J.W., Chun S.Y., Lee E.H., Ha Y.S., Lee J.N., Song P.H., Yoo E.S., Kwon T.G., Chung S.K., Kim B.S. (2019). Verification of mesenchymal stem cell injection therapy for interstitial cystitis in a rat model. PLoS ONE.

[3] Ho D.R., Chen C.S., Lin W.Y., Chang P.J., Huang Y.C. (2011). Effect of hyaluronic acid on urine nerve growth factor in cyclophosphamide-induced cystitis. Int. J. Urol..

[4] Kageyama A., Fujino T., Taki Y., Kato Y., Nozawa Y., Ito Y., Yamada S. (2008). Alteration of muscarinic and purinergic receptors in urinary bladder of rats with cyclophosphamide-induced interstitial cystitis. Neurosci. Lett..

[5] Fatima M., Anjum I., Abdullah A., Abid S.Z., Malik M.N.H. (2022). Boswellic Acids, Pentacyclic Triterpenes, Attenuate Oxidative Stress, and Bladder Tissue Damage in Cyclophosphamide-Induced Cystitis. ACS Omega.

[6] Tyagi P., Tyagi V., Yoshimura N., Witteemer E., Barclay D., Loughran P.A., Zamora R., Vodovotz Y. (2009). Gender-based reciprocal expression of transforming growth factor-β1 and the inducible nitric oxide synthase in a rat model of cyclophosphamide-induced cystitis. J. Inflamm..

[7] Haldar S., Dru C., Bhowmick N.A. (2014). Mechanisms of hemorrhagic cystitis. Am. J. Clin. Exp. Urol..

[8] Ribeiro R.A., Lima-Junior R.C., Leite C.A.V.G., Mota J.M.S.C., Macedo F.Y., Lima M.V., Brito G.A. (2012). Chemotherapy-induced hemorrhagic cystitis: Pathogenesis, pharmacological approaches and new insights. J. Exp. Integr. Med..

[9] Nasrin S., Masuda E., Kugaya H., Ito Y., Yamada S. (2013). Improvement by phytotherapeutic agent of detrusor overactivity, down-regulation of pharmacological receptors and urinary cytokines in rats with cyclophosphamide induced cystitis. J. Urol..

[10] Rooney P., Srivastava A., Watson L., Quinlan L.R., Pandit A. (2015). Hyaluronic acid decreases IL-6 and IL-8 secretion and permeability in an inflammatory model of interstitial cystitis. Acta Biomater..

[11] Xiao Y., Song Y.J., Song B., Huang C.B., Ling Q., Yu X. (2017). TGF-β/MAPK signaling mediates the effects of bone marrow mesenchymal stem cells on urinary control and interstitial cystitis after urinary bladder transplantation. Am. J. Transl.Res..

[12] He Y.Q., Zhang W.T., Shi C.H., Wang F.M., Tian X.J., Ma L.L. (2015). Phloroglucinol protects the urinary bladder via inhibition of oxidative stress and inflammation in a rat model of cyclophosphamide-induced interstitial cystitis. Chin. Med. J..

[13] Andersson M., Aronsson P., Doufish D., Lampert A., Tobin G. (2012). Muscarinic receptor subtypes involved in urothelium-derived relaxatory effects in the inflamed rat urinary bladder. Auton. Neurosci..

[14] Anjum I., Denizalti M., Kandilci H.B., Durlu-Kandilci N.T., Sahin-Erdemli I. (2017). Enhancement of S1P-induced contractile response in detrusor smooth muscle of rats having cystitis. Eur. J. Pharmacol..

[15] Yildiz S.Ç., Keskin C., Şahintürk V., Ayhanci A. (2020). Investigation of uroprotective effects of seed methanol extracts of Hypericum triquetrifolium Turra. on cyclophosphamide-induced bladder hemorrhagic cystitis and nephrotoxicity in Wistar albino rats. Çukurova Med. J..

[16] Gonçalves R.L.G., Cunha F.V.M., Sousa-Neto B.P.S., Oliveira L.S.A., Lopes M.E., Rezende D.C., Oliveira F.D.A. (2020). α-Phellandrene attenuates tissular damage, oxidative stress, and TNF-α levels on acute model ifosfamide-induced hemorrhagic cystitis in mice. Naunyn-Schmiedeb. Arch. Pharmacol..

[17] Leopoldini M., Pitarch I.P., Russo N., Toscano M. (2004). Structure, conformation, and electronic properties of apigenin, luteolin, and taxifolin antioxidants. A first principle theoretical study. J. Phys. Chem..

[18] Wang N., Yi W.J., Tan L., Zhang J.H., Xu J., Chen Y., Zhang R. (2017). Apigenin attenuates streptozotocin-induced pancreatic β cell damage by its protective effects on cellular antioxidant defense. Vitr. Cell. Dev. Biol..

[19] Singh J.P.V., Selvendiran K., Banu S.M., Padmavathi R., Sakthisekaran D. (2004). Protective role of Apigenin on the status of lipid peroxidation and antioxidant defense against hepatocarcinogenesis in Wistar albino rats. Phytomedicine.

[20] Zhang K., Kandhare A., Mukherjee-Kandhare A., Bodhankar S.L. (2020). Apigenin attenuated ethylene glycol induced urolithiasis in uninephrectomized hypertensive rats: A possible role of bikunin, BMP-2/4, and osteopontin. Pharmacogn. Mag..

[21] Altuntas C.Z., Daneshgari F., Sakalar C., Goksoy E., Gulen M.F., Kavran M., Qin J., Li X., Tuohy V.K. (2012). Autoimmunity to uroplakin II causes cystitis in mice: A novel model of interstitial cystitis. Eur. Urol..

[22] Vera P.L., Iczkowski K.A., Wang X., Meyer-Siegler K.L. (2008). Cyclophosphamide-induced cystitis increases bladder CXCR4 expression and CXCR4-macrophage migration inhibitory factor association. PLoS ONE.

[23] Korkmaz A., Topal T., Oter S. (2007). Pathophysiological aspects of cyclophosphamide and ifosfamide induced hemorrhagic cystitis; implication of reactive oxygen and nitrogen species as well as PARP activation. Cell Biol. Toxicol..

[24] Zhou X., Wang F., Zhou R., Song X., Xie M. (2017). Apigenin: A current review on its beneficial biological activities. J. Food Biochem..

[25] Toklu H., Alican I., Ercan F., Sener G. (2006). The beneficial effect of resveratrol on rat bladder contractility and oxidant damage following ischemia/reperfusion. Pharmacology.

[26] Nishijima S., Sugaya K., Kadekawa K., Ashitomi K., Ueda T., Yamamoto H. (2013). High-dose tranilast administration to rats creates interstitial cystitis-like symptoms with increased vascular permeability. Life Sci..

[27] Shabbir U., Anjum I., Naveed Mushtaq M., Nasir Hayat Malik M., Ismail S., Javed J., Rehman Z.U. (2022). Uroprotective and Hepatoprotective Potential of Anagallis arvensis against the Experimental Animal Model. J. Trop. Med..

[28] Assreuy A.M.S., Martins G.J., Moreira M.E.F., Brito G.A.C., Cavada B.S., Ribeiro R.A., Flores C.A. (1999). Prevention of cyclophosphamide-induced hemorrhagic cystitis by glucose-mannose binding plant lectins. J. Urol..

[29] Özgür A., Önol F.F., Ercan F., Tarcan T. (2008). Prophylactic role of oral L-Arginine on histological and contractile changes in a rat chronic bladder injury model. Urol. Int..

[30] Nyathi B., Bvunzawabaya J.T., Mudawarima C.V.P., Manzombe E., Tsotsoro K., Selemani M.A., Munyuki G., Rwere F. (2022). Antidiabetic and in silico molecular docking of *Xeroderris stuhlmannii* (Taub.) Mendonca EP Sousa phytochemical compounds on human α-glucosidases. bioRxiv.

[31] Rana S., Dixit S., Mittal A. (2019). In silico target identification and validation for antioxidant and anti-inflammatory activity of selective phytochemicals. Braz. Arch. Biol. Technol..

[32] Yende S.R., Shah S.K., Arora S.K., Moharir K.S., Lohiya G.K. (2021). In silico prediction of phytoconstituents from Ehretia laevis targeting TNF-α in arthritis. Digit. Chin. Med..

[33] Zahran E.M., Abdel-Maqsoud N.M., Tammam O.Y., Abdel-Rahman I.M., Elrehany M.A., Bakhsh H.T., Altemani F.H., Algehainy N.A., Alzubaidi M.A., Abdelmohsen U.R. (2022). Scabicidal Potential of Coconut Seed Extract in Rabbits via Downregulating Inflammatory/Immune Cross Talk: A Comprehensive Phytochemical/GC-MS and In Silico Proof. Antibiotics.

[34] Rahman M.H., Biswas P., Dey D., Hannan M.A., Sahabuddin M., Araf Y., Kwon Y., Emran T.B., Ali M.S., Uddin M.J. (2022). An In-Silico Identification of Potential Flavonoids against Kidney Fibrosis Targeting TGFβR-1. Life.

[35] Giglio D., Ryberg A.T., To K., Delbro D.S., Tobin G. (2005). Altered muscarinic receptor subtype expression and functional responses in cyclophosphamide induced cystitis in rats. Auton. Neurosci..

[36] Hegde S.S., Eglen R.M. (1999). Muscarinic receptor subtypes modulating smooth muscle contractility in the urinary bladder. Life Sci..

[37] Bonev A.D., Nelson M.T. (1993). ATP-sensitive potassium channels in smooth muscle cells from guinea pig urinary bladder. Am. J. Physiol. Cell. Physiol..

[38] Dela Peña I.C., Yoon S.Y., Kim S.M., Lee G.S., Ryu J.H., Park C.S., Kim Y.C., Cheong J.H. (2009). Bladder-relaxant properties of the novel benzofuroindole analogue LDD175. Pharmacology.

[39] Darblade B., Behr-Roussel D., Oger S., Hieble J.P., Lebret T., Gorny D., Benoit G., Alexandre L., Giuliano F. (2006). Effects of potassium channel modulators on human detrusor smooth muscle myogenic phasic contractile activity: Potential therapeutic targets for overactive bladder. Urology.

[40] Kishii K.I., Hisayama T., Takayanagi I. (1992). Comparison of contractile mechanisms by carbachol and ATP in detrusor strips of rabbit urinary bladder. JJP.

[41] Michel M.C. (2011). β-Adrenergic receptor subtypes in the urinary tract. Urin. Tract.

[42] Andersson K.E., Forman A. (1978). Effects of prostaglandins on the smooth muscle of the urinary tract. Acta Pharmacol. Toxicol..

[43] Shabbir R., Hayat Malik M.N., Zaib M., Alamgeer, Jahan S., Khan M.T. (2022). Amino Acid Conjugates of 2-Mercaptobenzimidazole Ameliorates High-Fat Diet-Induced Hyperlipidemia in Rats via Attenuation of HMGCR, APOB, and PCSK9. ACS Omega.

[44] O’Boyle N.M., Banck M., James C.A., Morley C., Vandermeersch T., Hutchison G.R. (2011). Open Babel: An open chemical toolbox. J. Cheminform..

[45] Najm S., Naureen H., Sultana K., Anwar F., Rehman S., Arshad S., Khan M.M. (2020). In-silico computational analysis of [6-(2, 3-Dichlorophenyl)-1, 2, 4-Triazine-3, 5-Diamine] metal complexes on voltage gated sodium channel and dihydrofolate reductase enzyme. Pak. J. Pharm. Sci..

[46] Yang J., Roy A., Zhang Y. (2013). Protein–ligand binding site recognition using complementary binding-specific substructure comparison and sequence profile alignment. Bioinformatics.

[47] Ebrahimipour S.Y., Sheikhshoaie I., Castro J., Dušek M., Tohidiyan Z., Eigner V., Khaleghi M. (2015). Synthesis, spectral characterization, structural studies, molecular docking and antimicrobial evaluation of new dioxidouranium (VI) complexes incorporating tetradentate N 2 O 2 Schiff base ligands. RSC Adv..

[48] Mahmoud W.H., Deghadi R.G., Mohamed G.G. (2020). Metal complexes of ferrocenyl-substituted Schiff base: Preparation, characterization, molecular structure, molecular docking studies, and biological investigation. J. Organomet. Chem..

[49] Kullmann F.A., Daugherty S.L., de Groat W.C., Birder L.A. (2014). Bladder smooth muscle strip contractility as a method to evaluate lower urinary tract pharmacology. J. Vis. Exp..

